# Identification of amendable risk factors for childhood stunting at individual, household and community levels in Northern Province, Rwanda – a cross-sectional population-based study

**DOI:** 10.1186/s12889-025-22329-8

**Published:** 2025-03-21

**Authors:** Albert Ndagijimana, Kristina Elfving, Aline Umubyeyi, Torbjörn Lind

**Affiliations:** 1 Department of Clinical Sciences, Pediatrics, Umeå University, Umeå, Sweden; 2 College of Medicine and Health Sciences, School of Public Health, University of Rwanda, Kigali, Rwanda; 3 School of Public Health and Community Medicine, Gothenburg University and The Queen Silvia’s Children Hospital, Gothenburg, Sweden

**Keywords:** Child, Community, Factors, Household, Individual, LMICs, Rwanda, Sub-Saharan Africa, Stunting, Undernutrition

## Abstract

**Background:**

Childhood stunting, defined as height-for-age below − 2 standard deviations (SD), disproportionately affects the Northern Province of Rwanda. We investigated risk factors contributing to stunting in this region at individual, household, and societal/community levels to inform future interventions.

**Methods:**

We conducted a population-based, cross-sectional study using a quantitative questionnaire in households with children aged 1–36 months in the Northern Province. Anthropometric measurements of children and mothers were taken to estimate nutritional status. Multivariable logistic regressions were performed to identify independent risk factors of stunting, reporting odds ratios, 95% confidence intervals and p-values.

**Results:**

Overall, stunting prevalence was 27.1% in children aged 1–36 months. At the individual level, boys exhibited 82% higher risk of stunting compared to girls (aOR: 1.82, 95% CI: 1.19, 2.78). Household-level factors such as maternal height and BMI were inversely associated with the risk of childhood stunting (aOR: 0.94, 95% CI: 0.90, 0.97 and aOR: 0.92, 95% CI: 0.86, 0.99, respectively). Other risk factors included no breastfeeding at the time of interview (aOR: 2.00, 95% CI: 1.23, 3.25), presence of twins or triplets aged 1–36 months (aOR: 2.60, 95% CI: 1.21, 5.57), female-headed (single parent) households (aOR: 2.07, 95% CI: 1.00, 4.26), and absence of handwashing facilities near the toilet (aOR: 3.30, 95% CI: 1.36, 7.98). No societal/community factors were significantly associated with childhood stunting in the Northern Province.

**Conclusion:**

Childhood stunting in the Northern Province of Rwanda is associated with several factors that could lend themselves to interventions, e.g., improved handwashing facilities, improved childcare practices and targeting vulnerable groups such as boys, households with twins or single parents. Additionally, a thorough exploration of identified risk factors through qualitative approaches involving all stakeholders in child and maternal nutrition is warranted.

**Supplementary Information:**

The online version contains supplementary material available at 10.1186/s12889-025-22329-8.

## Introduction

Stunting, i.e., length or height below − 2 standard deviations (SD) in comparison to international growth standards remains an imperative public health issue with 148.1 million cases globally, an estimated 22.3% of children under 5 in 2022 [1]. Most of these live in low- and middle-income countries (LMICs), where up to 64% are affected [1]. Its consequences are increased illnesses until adulthood, cognitive impairment and ultimately mortality, devastating families and communities and hampering long-term socioeconomic development [2].

While stunting prevalence has been declining steadily over the last decade, it remains elevated, across several low-income countries (LCI) in Asia and Africa. This lingering high prevalence is attributed to imbalances in efforts invested in its reduction. The current situation indicates a substantial gap in achieving the global target of reducing the number of children with stunting to 89 million by the year 2030 [1]. Thus, concerned and equitable efforts are imperative to address this challenge and align with the global objectives.

In LICs, childhood stunting incidence is the highest in the first three months of life and most growth faltering occurs before 24 months of age; underlining the importance of pre-pregnancy, prenatal and early life risk factors in its aetiology [3]. Previous studies, both those compiling data from multiple populations and those specifically from Rwanda, have identified individual risk factors such as small, or even average size at birth, male sex, age below 24 months and infections, e.g., diarrheal disease [4–6]. Household or family risk factors such as young maternal age, small maternal body size, low levels of education and media exposure, non-attendance of antenatal care and inadequate breastfeeding [4, 5], but also poverty, poor parental skills, lack of clean drinking water and limited access to improved toilet and waste disposal facilities have been acknowledged as well [7–10]. Lastly, factors at the community or societal level related to poor child growth were rural residence, lack of health care facilities and specific regional and geographical variations [4, 7–9].

In Rwanda, a few studies have tried to determine the risk factors of childhood stunting and many converge on those commonly reported risk factors of stunting in under five children described above [11]. Two previous studies have tried to disaggregate risk factors at the individual, maternal/household or community levels. In the first, Nshimyiryo et al. re-analysed the demographic and health survey (DHS) 2014-15 and identified family-level factors as the major causes of childhood stunting in Rwanda [6]. In the second, cross-sectional study, only poor households from five districts with high stunting prevalence were selected. This study identified handwashing practices, vegetable garden ownership, parental employment status and intimate partner violence as independently associated with childhood stunting [12]. At a provincial level, the Northern Province has, despite favourable agricultural circumstances, the highest prevalence of childhood stunting in the nation [13]. However, very little is known about individual, maternal/households/family and societal/community level risk factors, which would lend themselves to future interventions in that setting. In the present study, we used individual-level, cross-sectional data from this province to further investigate targets for stunting prevention. As the point of departure in selecting relevant variables in the analysis we have chosen the WHO conceptual framework on stunting prevention [14]. Our hypothesis was that stunting is the result of a complex series of events emanating from factors within the child, as well as in the household to which the child belongs and the community where the child lives. By applying multivariable analysis to the dataset, we could identify factors adaptable for community-based interventions.

## Materials and methods

### Study design and setting

We conducted a population-based, cross-sectional study with a quantitative household questionnaire on childhood nutritional status and maternal and child characteristics in selected households with eligible children in the Northern Province, one of the four provinces that together with the city of Kigali make up Rwanda in east-central Africa. The province has five districts: Burera, Gakenke, Gicumbi, Musanze and Rulindo; with 506,064 households and 2,038,511 habitants (15.4% of the Rwandan population) of whom 82.6% live in a rural setting. The National Institute of Statistics of Rwanda provided assistance through a comprehensive and up-to-date list of enumeration areas (EAs) or clusters (village in the Rwandan context).

### Study population and sampling

We targeted children below three years of age and the survey questionnaire was administered to the biological mother at a visit to the household. We excluded households with a mother younger than 18 years or when the mother was too unwell to respond to our questionnaire.

A sample of 615 households was obtained using the prevalence study sample size determination formula [15]. Then, we used two-stage cluster sampling to select households. The first stage consisted in randomly selecting 137 enumeration areas (villages) from the five districts of the Northern Province. The second stage consisted in randomly selecting 615 households with at least one child 1–36 months of age for the survey.

We collaborated with community health workers in charge of maternal and child health at village level who had an updated sampling frame, i.e., a list of households. From the list, systematic random sampling was done to have the required number of eligible households within a reasonable topographic distance between them for sufficient representation across the village.

### Data collection methods

We developed a structured household questionnaire that we administered to the target child’s biological mother to get data on individual characteristics of the participants, household sociodemographic characteristics (e.g., possessions, Ubudehe category, education, main occupation) and causes of undernutrition (e.g., source of drinking water, possession of livestock particularly, milking cows) and societal/community characteristics (e.g., distance to the nearest source of drinking water and health facility, knowledge of the existence of nutritional support programs in the community). Ubudehe is a Kinyarwanda term which represents a proxy for the household wealth, with four categories ranging from category 1, the poorest to category 4, the richest [16]. Anthropometric measurements were taken (length/height, weight, head circumference and mid-upper-arm circumference for children, height and weight for mothers) using locally produced length boards, digital weight scales (SECA AG, Hamburg, Germany) and tape measures. Geographical coordinates of the households were captured using GPS to measure the altitude (elevation). Data collection was performed by well-trained enumerators, university graduates from health sciences, after a one-week training in the city of Kigali and piloting in the Rulindo district of the study area (Northern Province).

### Study variables

To estimate the stunting prevalence in children aged 1–36 months as the outcome variable, we used the z-scores from the WHO Anthro software, with reference to the WHO child growth standards [17]. We defined Stunting as height-for-age (HAZ) below 2 standard deviations (SDs) and No stunting as HAZ > = 2 SDs, as per the Rwanda Demographic and Health Survey (RDHS) approach to estimate childhood nutritional status [13], the WHO child growth standards [18] and the WHO anthropometric measurement analysis [19].

The independent variables were classified at child’s individual, household and maternal levels. The child’s individual level characteristics included sex, age group in months, and health conditions in the two weeks preceding the survey (i.e., fever, cough, difficulty breathing, and presence of any chronic illness, defined by the mother). The choice of the variables for this study was informed by the WHO framework for childhood stunting [20], as well as the need by the Government of Rwanda; and we only considered those that could be amendable by evidence-based actions.

At household level, the maternal characteristics included age in years, weight in kilograms, height/stature in centimeters, age at marriage, and number of pregnancies. Household characteristics included variables grouped into childcare practices (whether the child was left alone more than 1 h in the preceding 15 days, left in care of another child during the preceding week, who prepared the meal for the child in the preceding 15 days, the number of days the child was fed by someone other than mother and father in the preceding two weeks, time to breast after birth, and whether the child was still breastfeeding at the time of the interview). Another group included household socioeconomic characteristics (maternal access to social support as a composite variable around having someone to assist in difficulties or problems, having someone to share food with in case of need, having a friend to assist when ill, having someone to lend money and being member of a cooperative; presence of siblings, particularly twins aged 1–36 months in the household, marital status, maternal education and main activity, Ubudehe category, sex of household head, family size, having health insurance). The intrahousehold food allocation group included having a milking cow or kitchen garden, the main source of food in the household, and household food security. As per Coates et al., the household food security access was measured using the Household Food Insecurity Access Scale (HFIAS), which is calculated using nine occurrence questions about food vulnerability or stress and coping behaviours with that stress, in terms of experience and the severity (frequency in the previous four weeks) [21]. Sanitation and water supply was measured by looking at the main source of drinking water, type of toilet and handwashing facilities and practices (if a handwashing station was situated within five meters from the toilet, if the mother had washed her hands with soap in the previous 24 h, or if the mother had washed her hands handwashing at critical moments, i.e., after helping a child defecate, before preparing food and after using the toilet), as well as water treatment prior to drinking.

### Statistical analysis

Based on the anthropometric measurements (weight in grams, height in centimeters) and age in months of the participating children, we computed the z-scores using WHO Antro version 3.2.2 and categorized them with reference to the WHO child growth standards [17] and WHO anthropometric measurements analysis [18]. After checking for and removing all implausible values (HAZ scores below − 6 SD or above + 6 SD), our outcome of interest, childhood stunting, was defined as height-for-age z-score (HAZ) below − 2 standard deviations (SD).

We describe all the continuous data with central tendency measures (means and standard deviations) depending on their distribution. All the categorical data are presented with frequencies and proportions. Part of the categorical variables were computed scores from a set of questions, like HFIAS and maternal access to social support.

Through bivariate logistic regression, we determined the relationship between each covariate and stunting. Then, all significant variables from the bivariate model were put into a full multiple logistic regression; and after adjusting for district, we identified the factors statistically significantly associated with childhood stunting in the Northern Province. For both bivariate and multiple logistic regression models, odds ratio (OR), 95% confidence interval (CI) and p-values are reported. Our statistical analysis was significant at 95% confidence level.

Finally, we intuitively built a Directed Acyclic Graph (DAG) [22] to illustrate variables for causation, mediation and confounding, from the multivariable full logistic model for childhood stunting in the Northern Province. DAGs are powerful and easy-to-learn tools to sharpen communication and guide the conduct of research, especially in clinical research [23]. We analyzed our data using Stata 18 (StataCorp. 2023. Stata: Release 18).

### Ethical considerations

Prior to collecting data, ethical approval was obtained from the University of Rwanda College of Medicine and Health Sciences Institutional Review Board (IRB) with Reference N^o^ 295/CMHS/IRB/2022, a unique approval for all eight doctoral students who are part of the multidisciplinary research project on child and maternal undernutrition in Northern Province of Rwanda. We also had the authorization from Ministry of Local Governance through the Ministry of Health, as well as the National Institute of Statistics of Rwanda Visa with Reference N° 0295/2021/10/NISR. Any information obtained from the respondents was kept confidential. Participation in the study was voluntary. Study participants did not have to respond to any questions where they were not feeling comfortable and could withdraw from the study at any time without any consequences.

## Results

### Participants’ characteristics and their association with childhood stunting

This study consisted of 601 mother-child pairs with the latter aged 1–36 months (Table 1). Overall, the stunting prevalence was 27.1%. The bivariate analysis found significantly higher risk of childhood stunting in boys, those aged 12–36 months. Mothers with 3–5 pregnancies had 42% lower risk than those with one or two pregnancies. Both maternal height and BMI were inversely associated with childhood stunting (Table 1).

**Table 1:** Background characteristics of participants based on stunting status and bivariate risk factors for stunting (*N* = 601)

Variables	Total (%)^a^	No stunting	Stunting	COR^b^	95% CI^c^	*P* for difference^d^
		*n* (%)	*n* (%)			
*Sex of child*						
Female	311 (51.7)	246 (79.1)	65 (20.9)	1.00		
Male	290 (48.3)	192 (66.2)	98 (33.8)	1.93	1.34, 2.79	< 0.001
*Age group (months)*						
Mean (SD)	17.7 (10)	15.9 (9.9)	22.4 (8.4)			
1–11	199 (33.1)	180 (90.4)	19 (9.6)	1.00		
12–23	213 (35.4)	144 (67.6)	69 (32.4)	4.54	2.61, 7.89	< 0.001
24–36	189 (31.4)	114 (60.3)	75 (39.7)	6.23	3.58, 10.86	< 0.001
*Fever in preceding 2 weeks* ^e^						
Yes	180 (30.2)	127 (70.6)	53 (29.4)	1.21	0.82, 1.78	0.347
*Cough in preceding 2 weeks* (*n* = *578*)^e^						
Yes	273 (47.2)	199 (72.9)	74 (27.1)	0.99	0.69, 1.44	0.977
*Difficulty breathing in preceding 2 weeks* (*n* = *573*)^e^						
Yes	131 (22.9)	89 (67.9)	42 (32.1)	1.39	0.91, 2.13	0.128
*Chronic illness* (*n* = *590*)^e^						
Yes	18 (3.1)	13 (72.7)	161 (27.3)	1.03	0.36, 2.92	0.962
*Maternal age (years)*						
Mean (SD)	32 (7.2)	32 (7.1)	32 (7.3)			
18–25	139 (23.2)	101 (72.7)	38 (27.3)	1.00		
25–35	269 (44.9)	198 (73.6)	71 (26.4)	0.95	0.60, 1.51	0.838
26–58	192 (32.0)	139 (72.4)	53 (27.6)	1.01	0.62, 1.65	0.957
*Maternal height (cm)*						
Mean (SD)	157.6 (6.1)	158.1 (6.0)	156.2 (6.0)	0.95	0.92, 0.98	0.001
*BMI* (*kg/m*^*2*^)^f^						
Mean (SD)	23.39 (3.33)	23.58 (3.30)	22.90 (3.35)	0.94	0.88, 0.99	0.028
*Age at marriage (years)* (*n* = *547*)						
Mean (SD)	22.2 (3.8)	22.2 (3.7)	22.3 (4.2)			
< 21	184 (33.6)	130 (70.6)	54 (29.4)	1.00		
21–24	244 (44.6)	190 (77.9)	54 (22.1)	0.68	0.44, 1.06	0.090
25–35	119 (21.8)	84 (70.6)	35 (29.4)	1.00	0.60, 1.66	0.990
*Number of pregnancies*						
1–2	279 (46.5)	194 (69.5)	85 (30.5)	1.00		
3–5	221 (36.8)	176 (79.6)	45 (20.4)	0.58	0.39,0.88	0.011
≥ 6	100 (16.7)	68 (68.0)	32 (32)	1.07	0.66,1.76	0.776

^a^Data are means and standard deviations (SD) or numbers and percentages. ^b^COR: crude odds ratio, ^c^95% confidence interval, ^d^logistic regression, ^e^reference category is no fever, no cough, no difficulty breathing, no chronic illness, ^f^body mass index

### Household, societal and community characteristics and their relationship to childhood stunting

Regarding feeding practices, maternal support and household characteristics, bivariate analysis showed an association between childhood stunting and delayed breastfeeding initiation, not being breastfed at the time of interview, being fed by someone other than one of the parents, maternal lack of social support, having a twin aged below 36 months in the household or households being food insecure, headed by a female (single parent household), lacking kitchen garden or poor water and hygiene facilities (only access to unimproved toilet facilities, lack of handwashing close to the toilet, not handwashing with soap or at critical moments) (Additional file 1). Of the societal and community factors, none was associated with a higher risk of stunting (Additional file 1).

### Independent risk factors associated with childhood stunting

The multivariable logistic regression showed that child’s sex and age, maternal height and BMI, breastfeeding at the time of interview, having twin and triple children aged 1–36 months in the household, having a female head of household (in practice a single parent household) and not having a place for handwashing within five meters from the toilet as factors statistically significantly associated with childhood stunting in the Northern Province. Because stunting was found to be spatially clustered across the five districts of the Northern Province in a previous study done as part of this project [24], we adjusted our analysis for the variable “district”. Also, the child’s age was removed from the model since it is obvious that stunting increases with increasing age of the child, especially in the first years of life.

After adjustment, boys had 82% higher risk of stunting than girls [adjusted odds ratio (aOR): 1.82, 95%CI: 1.19, 2.78]. Maternal body size was significantly associated with child growth: for every centimeter increase in the stature/length of the mother, the child was 6% less likely to be stunted (aOR: 0.94, 95%CI: 0.90, 0.97) and for every unit increase in the mother’s BMI, the child was 8% less likely to be stunted (aOR: 0.92, 95%CI: 0.86, 0.99). who were no longer breastfeeding had twice as high risk of stunting than those who were breastfeeding at the time of interview (aOR: 2.00, 95%CI: 1.23, 3.25). Children who were twin or triplet siblings had 2.6 times higher odds of stunting than single born children (aOR: 2.60, 95%CI: 1.21, 5.57). Female-headed households had 2.07 times higher odds of childhood stunting than male-headed ones (aOR: 2.07, 95%CI: 1.00, 4.26). Households without a place for handwashing within five meters from the toilet had 3.30 times higher odds than those with it (aOR: 3.30, 95%CI: 1.36–7.98) (Table 2).

**Table 2:** Multivariable analysis for factors associated with childhood stunting in the Northern Province, Rwanda

Variables	Unadjusted full model			Adjusted full model		
	OR	(95% CI)	p-value	aOR	(95% CI)	p-value
***Child individual factors***						
Sex of the child						
Girl	1.00			1.00		
Boy	1.84	1.22, 2.76	0.003	1.82	1.19, 2.78	0.006
***Household factors (maternal)***						
Maternal height	0.94	0.91, 0.97	0.001	0.94	0.90, 0.97	0.001
Maternal BMI	0.93	0.87, 1.00	0.037	0.92	0.86, 0.99	0.028
Number of pregnancies						
1–2	1.00					
3–5	0.64	0.40, 1.02	0.063			
6 or more	1.10	0.62, 1.97	0.735			
***Household factors (childcare practices)***						
Days the child was fed by someone other than mother and father in the last two weeks						
Never	1.00			1.00		
1 day or more	1.54	1.00, 2.37	0.049	1.51	0.97, 2.36	0.069
How long after birth the mother first put the baby to the breast						
Within 1 h	1.00					
After 1 h or more	1.41	0.77, 2.59	0.266			
Child still breastfeeding at the time of the interview						
Yes	1.00			1.00		
No	1.90	1.19, 3.04	0.007	2.00	1.23, 3.25	0.005
***Household factors (socioeconomic characteristics)***						
Mother receives societal support						
Yes	1.00					
No	1.57	0.99, 2.47	0.054			
The child has a twin or triplet sibling						
No	1.00			1.00		
Yes	2.47	1.16, 5.26	0.019	2.60	1.21, 5.57	0.014
Sex of household head						
Male	1.00			1.00		
Female	1.66	0.84, 3.30	0.147	2.07	1.00, 4.26	0.049
***Household factors (intrahousehold food allocation)***						
Total Score	0.99	0.94, 1.05				
Household Food Insecurity Access						
Food secure	1.00			1.00		
Mildly Food Insecure	2.05	0.75, 5.64	0.164	2.45	0.87, 6.93	0.091
Moderately Food Insecure	1.36	0.60, 3.05	0.463	1.40	0.60, 3.27	0.445
Severely Food Insecure	1.57	0.69, 3.56	0.277	1.66	0.71, 3.92	0.246
***Household factors (sanitation and water supply)***						
Type of latrine/toilet						
Improved	1.00					
Non improved	0.93	0.60, 1.42	0.726			
Handwashing facility within 5 m from the toilet/latrine						
Yes	1.00			1.00		
No	3.33	1.41, 7.91	0.006	3.30	1.36, 7.98	0.008
Mother has been able to wash with soap and water in the last 24 h						
Yes	1.00					
No	1.41	0.80, 2.47	0.231			
Handwashing at critical moments						
Yes	1.00					
No	1.14	0.72, 1.79	0.580			

### Directed acyclic graph for childhood stunting in the Northern Province, Rwanda

**Fig. 1:**
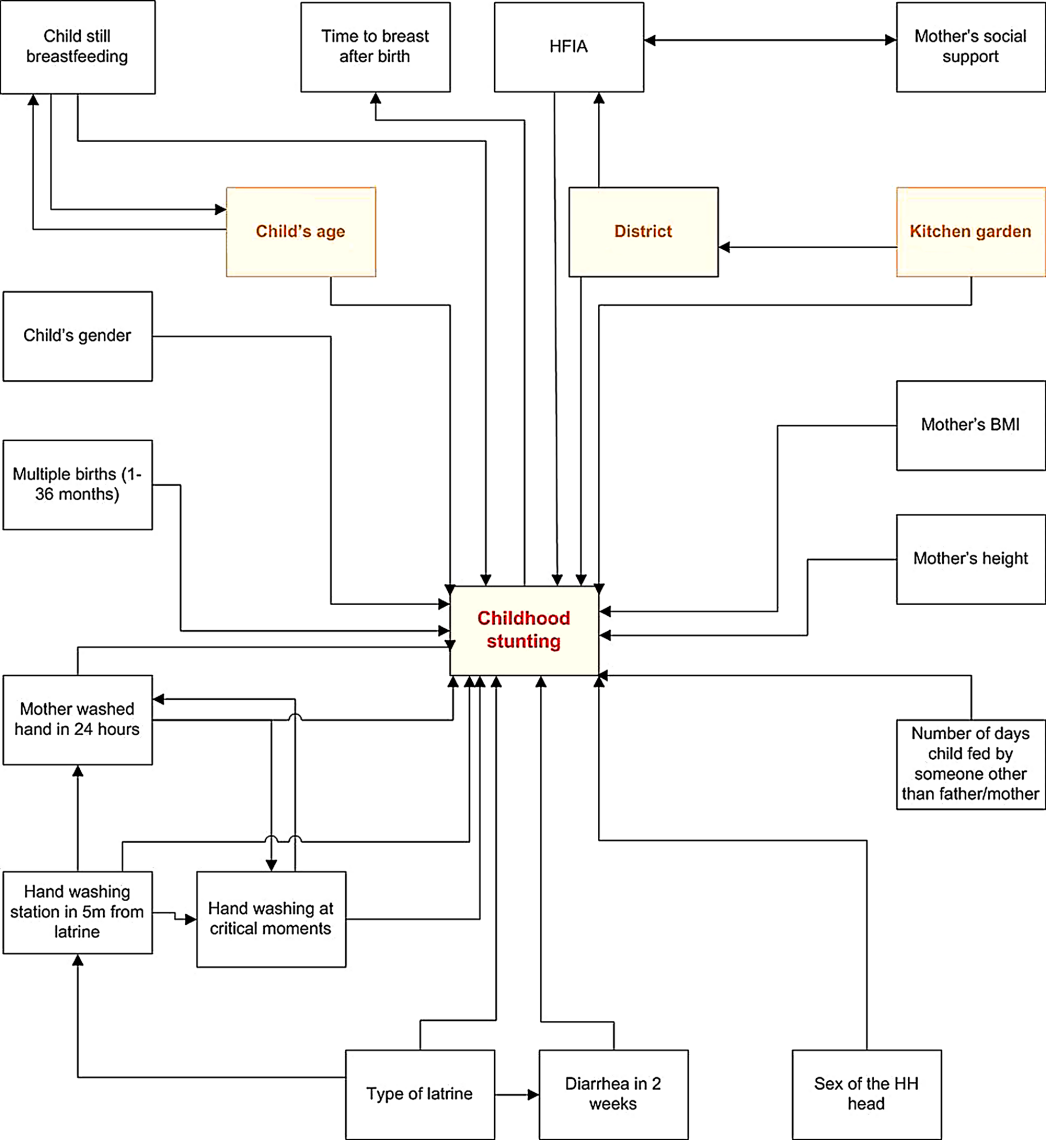
Directed Acyclic Graph for childhood stunting, Northern Province, Rwanda, 2021. Arrow shows the association between variables, childhood stunting is the outcome, highlighted variables are confounders or mediators

As our DAG illustrates, all the variables came out as exposures, except kitchen garden, district of residence and child’s age in months. It is already known that child’s age in months was thought of as confounding the association of child’s status about breastfeeding on stunting. The HFIA (especially the availability of food for household consumption) influenced the risk of stunting, but it relied on district. Further, childhood stunting was influenced by geographical variability (our own observation concerning the geographical clustering of childhood stunting) [24]. Given their confounding patterns, both district and kitchen garden were removed from the full model adjustment. The child’s age was not considered given prior knowledge of its association with childhood stunting (from our analysis, it influenced the model, yet it is difficult to account for during intervention, rather than focusing our intervention on geographical variability). Two variables (type of toilet, handwashing at critical moments) were seen as mediating the effect of other variables on childhood stunting (Fig. 1). We highlight that all the variables in the DAG had a statistically significant relationship with childhood stunting at initial phase (bivariate logistic regression), suggesting a causal link with the outcome.

## Discussion

The study aimed to determine amendable risk factors for childhood undernutrition at individual, household and community levels in the Northern Province of Rwanda [20]. Our analysis identified several such variables. As risk factors with the potential to be affected through future interventions in the study area we want to discuss the sex of the child, maternal height and BMI, breastfeeding practices and household composition and sanitation.

At the child individual level, our finding that boys had 82% higher odds of stunting than girls confirm the results from other studies conducted in Rwanda showing that male children are at higher risk of stunting than females [25–30], especially in Musanze [31] and Gicumbi districts of the Northern Province [32]. In view of the limited knowledge on sex differences as an influence of childhood nutrition, several studies have tried to explain the complex interaction of social, environmental and genetic patterns, which are more pronounced in socio-economically deprived communities leading to boys being more exposed than girls [33]. The Northern Province is entirely hilly with higher rates of infections in children under five than other provinces, including severe acute respiratory illness (SARI) hospitalization [34]. Also, the RDHS 2019-20 reported a higher national prevalence on ARI in boys than girls [13]. This highlights the need for enhanced childcare practices for protection against infections. These could take account of feeding practices including enhanced supplementation and growth monitoring to improve immunity [2].

At the household level, maternal body size was inversely associated with childhood stunting. This agrees with other studies conducted in Rwanda, where maternal height is strongly associated with childhood stunting [6, 29, 35, 36], as well as her weight [25]. Intergenerational effects of childhood malnutrition are well documented, whereby intrauterine and infant growth failure are reported to associate with low birth weight and later with childhood stunting [37]. Short mothers are more likely to have stunted children and the odds of childhood stunting decrease as maternal BMI increases [38]. The maternal height influence on childhood growth is visible in offspring with low birth weight and later by the age of two years, which clearly depicts the importance of nutrition during adolescence and pregnancy to cut the intergenerational cycle of undernutrition [37].

Children who were not breastfed at the time of the survey had two times higher risk of stunting compared to children of the same age being breastfed. A re-analysis of the Rwanda DHS 2014-15 found likewise that children who were being breastfed at the time of the survey had 98% less chance of stunting than those who were not [28]. Also, in Burundi, a neighbouring country to Rwanda and in the same economic development category but with even higher prevalence of childhood stunting, breastfeeding continuation up to two years was found to protect children against stunting [39]. However, previous breast-feeding interventions have not impacted growth or body composition in any substantial way [40]. It should be noted that the studies included in the meta-analysis did not test breastfeeding per se but interventions to improve breastfeeding rates. The finding that observational studies indicate an association between breastfeeding and the risk of stunting but that interventions find no effect poses a conundrum where it is prudent to ask what information data on current breastfeeding status provides. At present, promotion of exclusive breastfeeding for the first six months of life and continued breastfeeding for two years has many health benefits in most populations, but further studies are needed to show if improved growth is among them [41]. Going beyond up to 36 months revealed beneficial effect, which could be considered as a point of emphasis during nutrition education sessions in health facilities and the community.

In our study, the risk of stunting were 2.58 times higher in children who were twin or triplets aged 1–36 months in the same household compared to single born children. DHS re-analyses from a number of sub-Saharans countries, report that multiple births constitute a twice increased risk of stunting [30]. Possible mechanisms explaining the risk of multiple births include increased risks of low birth weight and premature birth, themselves risk factors for childhood undernutrition, demands of breast milk beyond what the mother can provide and competition for resources including nutrients between the siblings [42]. Multiple births are relatively rare, 6.5% of the households in the present sample had twins. Thus, it would be relatively easy to identify these families and offer targeted support, including other means of infant nutrition with nutritional properties similar to breast milk.

Female-headed households reported 2.07 times higher odds childhood stunting than male-headed households. A study in selected areas of Rwanda with lower food security reported that female-headed households had 2.2 times higher the odds of childhood stunting that male-headed households [43]. Similarly, a study from Burundi reported that mothers living alone, i.e., heading their households had 1.5 time higher the odds of childhood stunting than those living in as a couple [39]. In patriarchal communities such as those in the study area, female-headed households, which in practice are single parent households, may experience prejudices and low social support contributing to financial hardships and difficulties in achieving prolonged breastfeeding and providing adequate complementary foods. A further analysis of our data found that 74.5% of female heads of households did not receive social support from the community (not reported). Again, single-parent households are relatively uncommon, 5.6% in the present study, and with procedures for identification in place, they would lend themselves to support.

Insufficient handwashing practices, particularly not having a place for handwashing close to the latrine was associated with a 3.28 higher risk of stunting. A previous study among poor households in Rwanda showed that good handwashing practices are associated with 82% less chance of childhood stunting [12]. Mothers, the main handlers of children can act as vectors for contamination between the toilet and the children without proper hygienic facilities after using the toilet or cleaning their children, which in turn can cause infections and contribute to poor growth [44]. Lack of sufficient amounts of clean water remains an issue in many communities of Rwanda, and very limited amounts are used at critical moments, such as when drinking and preparing meals. This was evident in the Rwandan DHS from 2019 to 20 which found 84% of the households with a place for handwashing (79.2% in the Northern Province), but with 41% of them with water available, and only 32% with soap or another cleansing agent available at the time of observation [13]. However, randomized interventions with improved WASH standards have had no effects on child growth [2, 45], leaving the present findings somewhat in limbo putting into question what measures of inadequate circumstances of water supply and washing facilities indeed measure.

Finally, household food insecurity was associated with childhood stunting in the bivariate analysis but did not reach the statistical significance in the multivariable analysis. This was probably due to the low level of significance in the bivariate analysis, whereby one category was not significant at all, which was completely lost during adjustment. The variation of food insecurity in the study area was low with almost eight out of ten households reporting moderate or severe food insecurity. In Gicumbi district, Northern Province, a previous study found higher odds of severe stunting among children from households with moderate and severe food insecurity compared with those from food secure households [46]. In Burundi, the odds of stunting were reported to increase with the severity of food insecurity, an exact same pattern as the Northern Province of Rwanda [39]. Lastly, recent a meta-analysis has investigated association between food security and stunting, showing a modestly increased risk of 14% among children < 5 years of age [47]. The Northern Province has from an agricultural point of view beneficial circumstances to produce foods of high quality that could serve as basis for a good complementary diet. Provisions of more diversified and nutrient rich complementary foods and improved knowledge on complementary feeding practices have provided low to moderate level evidence that anthropometric measurements can be improved in food insecure settings [48]. More research is needed on what complementary food strategies, including types of foods, their age of introduction and by what schedule, work best in study area.

### Strengths and limitations

As strengths, the present study is the first of its kind to analyze amendable risk factors of childhood stunting with adjustment for district after it was found that stunting is clustered in the study area [24]. Many previous studies have re-analyzed Rwandan DHS data, but in the present, we selected representative sample of the population in a defined setting, recruited participants using sound methodology, giving the study high external validity in similar settings. The study considered child, mother, household, and societal/community levels to determine associated factors, which could trigger level-specific and level-bridging interventions. Lastly, this study was analytical, showing factors statistically associated with childhood stunting in the Northern Province of Rwanda, which could guide future interventions.

Cross-sectional study designs have several strengths including low costs in comparison to the plethora of data that can be collected at one time point and that multiple outcomes and exposures can be studied at once. However, the design does not allow for investigations of temporal effects, which in studies of growth outcomes can be a limitation and it is also difficult to draw causal inferences. In the present study, we pick up risk factors as significant, also in the multivariable model, where previous interventions have not proven causality, e.g., the associations between continued breastfeeding or WASH practices and stunting. This distorts the conclusions we can draw. Also, we were not able to collect physical samples in order to investigate the composition of water that is used at household level, nor collect data on environmental composition that could influence the child growth, as done by other studies [10]. Mothers less than 18 years of age were excluded from the study, due to lack of maturity age to consent in Rwanda, yet they are more vulnerable through their often single-parent household status and having their children neglected when they stay in their parents’ house, with a possibility of increasing the family size, a commonly known risk factors of childhood stunting. We think their exclusion did not considerably affect the validity of the study findings, since teenage pregnancy (among 15–19 years old girls) was estimated at 5% as per the most recent RDHS [13]. Lastly, we excluded primary caregivers who were not biological parents. However, since many children 1–36 months have biological mothers as their primary caregivers, we believe this did not affect the validity of our findings to a considerable extent.

## Conclusion

In this study, we aimed to determine amendable risk factors at individual, household, and community level in the study area. We were able to identify several independent factors at individual and household levels, i.e., the sex of the child, maternal size, child feeding practices, family composition, particularly the presence of siblings of the same age and washing facilities, but no community level risk factors were found. We conclude that childhood stunting stands out as a phenomenon with complex links between its covariates, mediators and confounders. For the time being, interventions could include caregiver’s education on childcare practices, with a certain focus on boys, nutritional education during pregnancy, education on breastfeeding continuation until the age of 36 months, special attention on twins or triplets, special support or economic empowerment for female-headed households with young children, as well as effective handwashing practices for caregivers.

The results have generated a number of possible hypotheses as to the causes of stunting, but further studies, both qualitative and also intervention studies are needed for comprehensive and holistically informed actions in the Northern Province of Rwanda.

## Electronic supplementary material

Below is the link to the electronic supplementary material.

Supplementary Material 1

Supplementary Material 2

## Data Availability

The datasets generated and/or analyzed during the current study are not publicly available due to privacy of the participants (their personal identity and other very sensitive information. The big dataset hosts records from eight multidisciplinary projects, including gender-based violence and mental health) but are available from the corresponding author upon reasonable request.

## Abbreviations

BMI: Body Mass Index
CI: Confidence Interval
DAG: Directed Acyclic Graph
DHS: Demographic and Health Survey
EA: Enumeration Area
GPS: Global Positioning System
HAZ: Height-for-Age z-score
HFIAS: Household Food Insecurity Access Score
IRB: Institutional Review Board
LMICs: Low- and middle-income countries
OR: Odds ratio
SD: standard deviation
WASH: Water, hygiene and sanitation
WHO: World Health Organization
