# Supplementary material for: Identification of amendable risk factors for childhood stunting at individual, household and community levels in Northern Province, Rwanda – a cross-sectional population-based study
Source: BMC Public Health. 2025 Mar 21;25:1087. doi: 10.1186/s12889-025-22329-8 (PMC11927283; doi:10.1186/s12889-025-22329-8)
Supplement: Supplementary file 1 — Supplementary Material 1 [file 12889_2025_22329_MOESM1_ESM.pdf]

New questionnaire:

a0_name_of_interviewer	Name of interviewera	
a0_date_of_interview	Date of interview	
a0_phone_address	Phone number and/or contact address of respondent:	
a0_guardian_signed_witnessed_consent	Has the respondent signed a witnessed consent?	
a0_respondent	Is the respondent the mother of the household?	
a0_ubedehe_category	Ubedehe category?	
a1_age	A 1. What is your age (in years) (addressed to the mother)?	
a1_age_dont_know	do not know	false
a2_deliver_alive_child_in_time_period	A 2a. Is there a child in this household with an age between 1 month up to 3 years that is still alive?	
a2_twin_or_triple	A 2b. If yes in A 2a, is the child a twin or a triplet?	
a3_1_respondent_names	Respondent names	
a3_2_farm_id	Farm/household ID	
a3_4_district	District	
a3_5_cell	Cell	
a3_6_sector	Sector	
a3_7_village	Village	
a3_8_latitude	Latitude	
a3_9_longitude	Longitude	
a4_maritial_status	A 4. What is your marital status?	
a5_married_at_age	A 5. At what age did you get married?	
a6_number_people_in_household	A 6. How many people do most of the time live in your household?	
a7_number_children_birth_to	A 7. How many children did you give birth to?	
a8_number_alive_children	A 8. How many are alive today?	
a9_children_ages	A 9.How old are they (in years)?	
a10_sex_of_household_head	A 10. What is the sex of your head of household?	
a11_relationship_with_household_head	A 11. What is your relationship with the household head?	
a12_religion	A 12. What is your religion?	
a13_attended_school	A 13. Have you ever attended school?	
a14_education_level	A 14. What level of education did you reach?	
a15_total_years_school_and_university	A 15. Total number of years at school and university level	
a16_main_daily_activity	A 16. What is your main daily activity?	

a17_paidjob_type	A 17. If you have a paid job, are you in… ?	
a18_occupation	A 18. What is your occupation?	
a19_health_insurance	A 19. Do you have a health insurance? (Report the main one)	
a20_partner_age	A 20. Age of your husband/partner (in years)	
a21_partner_attended_school	A 21. Has your husband/partner ever attended school?	
a22_partner_education_level	A 22. What level of education did your husband/partner reach?	
a23_paertner_total_years_school_university	A 23. Total number of years at school and university level	
a24_partner_has_job	A 24. Does your husband/partner have a paid job?	
a25_partner_job_type	A 25. If he has a paid job, is he in?	
a26_partner_occupation	A 26. What is your husband/partner's occupation?	
a27_total_household_income	A 27. What is the total household income per month? (This should be the sum of what all household members bring in taken together)	
a29_1_food	1. Food	
a29_2_health_care	2. Health care	
a29_3_rent	3. Rent	
a29_4_education	4. Education	
a29_5_transport	5. Transport	
a29_6_investment	6. Investments	
a29_7_saving	7. Savings	
a29_8_loans	8. Loans	
a30_sell_household_assets_last month	A 30. In the past month, did you have to sell any household assets? If so for what reason?	
a31_house_type	A 31. What type of house do you live in?	
a32_drinking_water_source	A 32. What is the main source of drinking water for members of your household?	
a33_how_long_take_get_water	A 33. How long does it take to get water, and come back including queueing? (Go, queue and return)	
a34_treat_water_to_drink	A 34. Do you treat water to drink in any way?	
a35_how_treat_drinking_water	A 35. What are you using to treat drinking water?	
a36_water_used_inday_for_hygiene_drinking	A 36. How many liters of water do you use in a day for hygiene and drinking?	
a38_kind_of_toilet	A 38. What kind of toilet facility does your household have?	
a39_share_toilet_with_other_families	A 39. Do you share toilet facilities with other families?	
a40_how_many_household_use_this_toilet	A 40. How many households use this toilet facility?	
a41_have_able_wash_with_soap_last_24h	A 41. Have you been able to wash with soap and water in the last 24 hours?	
a42_handwashing_place_near_toilet	A 42. Is there a place for handwashing near (within 5 m) from the toilet/latrine?	
a43_what_done_to_dispose_stools	A 43. The last time (your child) passed stools, what was done to dispose of the stools?	
a44_do_wash_hands_after_helping_child_defecate	A 44. Do you wash your hands after helping your child defecate?	
a45_washing_hand_before_preparing_food	A 45. Do you wash your hands before preparing food?	
a46_wahsing_hand_after_toilet	A 46. Do you wash your hands after using the toilet?	
a47_cocking_done_inside	A 47. Is your cooking done inside the house, outside or both?	
a48_access_to_electricity	A 48. Dose your household have access to electricity?	
a49_bic_mot_car	A 49. Does your household have:	
a50_appliances	A 50. Does any member/s of your household have:	
b1_have_friend_to_assist_when_ill	B 1. Do you have a friend or family member that will assist you if you become ill?	
b2_share_food_with_you	B 2. Share food with you?	

b3_share_their_house_with_you	B 3. Share their house with you?	
b4_lend_you_money	B 4. Lend you money	
b5_help_guidance_in_problems	B 5. Help you with guidance to improve your situation when you have problems?	
b6_support_in_personal_problems	B 6. Offer support to you if you run into personal problems?	
b7_belong_to_any_association	B 7. Do you belong to any association, such as a cooperative, church group, women's group, youth group, sports organization?	
c1_number_of_pregnancy	C 1. How many times have you been pregnant?	
c2_alive_born	C 2. Number of children born alive?	
c3_born_year_weight	C 3. What years were they born (live births) and what were their birth weights (in grams)?	
c4_children_stillborn	C 4. Number of children that were stillborn?	
c5_boys	C 5. How many of your children are boys?	
c6_girls	C 6. How many of your children are girls?	
c7_number_born_caesarean	C 7. How many of your children were born by caesarean section?	
c8_number_born_at_health_facility	C 8. How many of your children were born at a health facility?	
c9_number_miscarriages	C 9. Number of miscarriages?	
c11_number_anc_visits	C 11. How many times did you go to the health centre for Antenatal care (ANC)-visits during your pregnancy with the child NAME?	
c13_where	C 13. Did you go anywhere else for antenatal care (ANC) check-ups during your pregnancy?	
c15_where_give_birth	C 15. Where did you give birth?	
c16_mode_of_delivery	C 16. Mode of delivery?	
d0_child_name	What is child's name?	
d1_birthday_day	Day	
d1_birthday_month	Month	
d1_birthday_year	Year	
d2_child_age	D 2. How old is (NAME) (in month)?	
d3_birth_certificate	D 3. Does (NAME) have a birth certificate?	
d4_birth_been_registered	D 4. Has (NAME)'s birth been registered with *THE CIVIL AUTHORITIES*?	
d5_know_howto_register	D 5. Do you know how to register (NAME)'s birth?	
d7_child_gender	D 7. Is it a boy or girl/(Sex of child)	
d8_birthweight	D 8. Birthweight (g)	
d9_weight	1. Weight of the child in grams	
d9_weight of mother	1.1_weight of the mother in Kg	
d9_height	2. Height/Length of the child	
d9 _height of mother	2.1 height or length of the mother	
d9_head_circumference	3. Head circumference	
d9_mid_upper_arm_circumference	4. Mid Upper Arm Circumference:	
d9_temperature	5. Temperature? (one decimal)	
d9_respiratory_rate	6. Respiratory rate ? (must record 2 numbers XY)	
d9_haemoglobin	7. Haemoglobin result today (one decimal)	

d9_sample_collection_rectal_swab_performed	8. Sample collection- rectal swab sample collection from the child NAME performed	
d9_study_id	9. Indicate the study-ID	
d10_bipedal_edema	D 10. Does the child have swollen/edematous legs?	
d10_bipedal_edema_both_legs	Are both legs swollen/edematous?	
d11_visible_severe_wasting	D 11. Does the child have visible severe wasting, i.e. Is the child too thin?	
d12_weight_for_height	D 12. What is the Weight-for-height (W/H) of the child?	
d13_child_overal_health	D 13. Would you say that the overall health of the child today is	
d15_days_left_alone_more_than_hour	D 15. Left alone for more than 15 minutes?	
d16_days_left_with_another_child	D 16. Did you have to leave your child in the care of another child, that is, someone less than 10 years old, for more than an hour during the last week?	
d17_read_books	D 17. Read books or looked at picture books with (name)?	
d18_told_stories	D 18. Told stories to (name)?	
d19_sang_songs	D 19. Sang songs to or with (name), including lullabies?	
d21_played_with	D 21. Played with (name)?	
d22_named_counted_draw_things_with	D 22. Named, counted, or drew things for or with (name)?	
d23_can_pickup_small_object	D 23. Can (name) pick up a small object with two fingers, like a stick or a rock from the ground?	
d26_have_difficulty_walking	D 26. Does (name) have difficulty walking?	
d27_uses_equipment_for_walking	D 27. Does (name) use any equipment or receive assistance for walking?	
d29_has_difficalty_understanding_you	D 29. Does (name) have difficulty understanding you?	
d30_when_speeks_you_have_difficalty_understanding	D 30. When (name) speaks, do you have difficulty understanding (him/her)?	
d32_compared_to_other_has_difficalty_playing	D 32. Compared with children of the same age, does (name) have difficulty playing?	
d33_compared_to_other_how_much_hit_other	D 33. Compared with children of the same age, how much does (name) kick, bite or hit other children or adults?	
d34_has_any_vaccinations	D 34. Did (NAME) ever have any vaccinations to prevent him/her from getting diseases, including vaccinations received in a national immunization day campaign?	
d35_has_national_immunisation_record	D 35. Do you have a National Child Immunisation Record or immunisation records from a private health provider or any other document where (NAME)'s vaccinations are written down?	
d38_is_child_ill_now	D 38. Is the child ill now?	
d40_oral_intake	D 40. Oral intake?	
d41_vomiting	D 41. Vomiting?	
d42_ear_discharge	D 42. Ear discharge?	
d43_antibiotic_last_2weeks	D 43. Antibiotic use in the last two weeks?	
d45_diarrhea_in_last_2weeks	D 45. Has name suffered from diarrhea in the past two weeks	
d46_blood_in_stool	D 46. Was there blood in the stool at any time?	
d47_given_child_during_diarrhea	D 47. What was given to the child to drink during the time s/he had diarrhea?	
d52_fever_in_last_2weeks	D 52. At any time in the last two weeks, has (name) been ill with a fever?	
d53_during_illness_has_blood_testing	D 53. At any time during the illness, did (name) have blood taken from (his/her) finger or heel for testing?	
d54_illness_with_cough_last_2weeks	D 54. At any time in the last two weeks, has (name) had an illness with a cough?	

d55_difficulty_breathing_last2weeks	D 55. At any time in the last two weeks, has (name) had fast, short, rapid breaths or difficulty breathing?	
d57_take_child_clinic_by	D 57. IF your child has an illness that you cannot manage at home on your own and you need to seek health care. What kind of transportation do you use? TRAVEL BY:	
d58_hours	Hours	
d58_minutes	MINUTES	
d59_seek_advice_from_other_sources	D59. Did you seek any advice or treatment for the illness (diarrhea, cough or fever) from any source?	
d60_1_public_medical_sector	PUBLIC MEDICAL SECTOR	
d60_2_private_medical_sector	PRIVATE MEDICAL SECTOR	
d60_3_dk_public_or_private	Don't know PUBLIC OR PRIVATE	
d60_4_other_source	OTHER SOURCE	
d60_5_other_specify	OTHER (specify)	
d60_6_dk	DON'T KNOW / DON'T REMEMBER	false
d61_child_has_chronic_illness	D 61.Does your child have any of these diseases?	
d62_child_take_medicine_everyday	D 62. Does the child take a medicine every day?	
d63_which_medicine	D 63. If yes, which medicine?	
e1_worried_have_enough_food	E 1. In the past four weeks, did you worry that your household would not have enough food?	
e2_not_eat	E 2. In the past four weeks were you or any household member not able to eat the kinds of foods you preferred because of a lack of resources?	
e3_limited_food	E 3. In the past four weeks, did you or any household member have to eat a limited variety of foods due to a lack of resources?	
e4_eat_food_unwanted	E 4. In the past four weeks, did you or any household member have to eat some foods that you really did not want to eat because of a lack of resources to obtain other types of food?	
e5_eat_smaller_meal	E 5. In the past four weeks, did you or any household member have to eat a smaller meal than you felt you needed because there was not enough food?	
e6_fewer_meal_in_a_day	E 6. In the past four weeks, did you or any other household member have to eat fewer meals in a day because there was not enough food?	
e7_no_food_to_eat	E 7. In the past four weeks, was there ever no food to eat of any kind in your household because of lack of resources to get food?	
e8_goto_sleep_hungry	E 8. In the past four weeks, did you or any household member go to sleep at night hungry because there was not enough food?	
e9_no_eating_day_and_night	E 9. In the past four weeks, did you or any household member go a whole day and night without eating anything because there was not enough food?	
e11_borrow_food	E 11. In the past four weeks did you or any household member borrow food or rely on help from a relative or friend?	
e13_mother_female_guardian_weight	E 13. Weight of the mother	
e14_1_1	Breakfast	
e14_1_2	Snack	
e14_1_3	Lunch	
e14_1_4	Snack	

e14_1_5	Dinner	
e14_1_6	Snack	
e14_1_f1	a. CEREALS	false
e14_1_f1_times_yesterday	How many times yesterday (past 24 hours)?	
e14_1_f1_source	Main source of food	
e14_1_f2	b. WHITE ROOTS AND TUBERS	false
e14_1_f2_times_yesterday	How many of times yesterday (past 24 hours)?	
e14_1_f2_source	Main source of food	
e14_2_f1	c. LEGUMES, NUTS AND SEEDS	false
e14_2_f1_times_yesterday	How many of times yesterday (past 24 hours)?	
e14_2_f1_source	Main source of food	
e14_3_f1	d. VITAMIN A RICH VEGETABLES AND TUBERS	false
e14_3_f1_times_yesterday	How many of times yesterday (past 24 hours)?	
e14_3_f1_source	Main source of food	
e14_3_f2	e. DARK GREEN LEAFY VEGETABLES	false
e14_3_f2_times_yesterday	How many of times yesterday (past 24 hours)?	
e14_3_f2_source	Main source of food	
e14_3_f3	f. OTHER VEGETABLES	false
e14_3_f3_times_yesterday	How many of times yesterday (past 24 hours)?	
e14_3_f3_source	Main source of food	
e14_4_f1	g. VITAMIN A RICH FRUITS	false
e14_4_f1_times_yesterday	How many of times yesterday (past 24 hours)?	
e14_4_f1_source	Main source of food	
e14_4_f2	h. OTHER FRUITS	false
e14_4_f2_times_yesterday	How many of times yesterday (past 24 hours)?	
e14_4_f2_source	Main source of food	
e14_5_f1	i. ORGAN MEAT	false
e14_5_f1_times_yesterday	How many of times yesterday (past 24 hours)?	
e14_5_f1_source	Main source of food	
e14_5_f2	j. FLESH MEATS	false
e14_5_f2_times_yesterday	How many of times yesterday (past 24 hours)?	
e14_5_f2_source	Main source of food	
e14_5_f3	k. EGGS	false
e14_5_f3_times_yesterday	How many of times yesterday (past 24 hours)?	
e14_5_f3_source	Main source of food	
e14_5_f4	l. FISH	false
e14_5_f4_times_yesterday	How many of times yesterday (past 24 hours)?	
e14_5_f4_source	Main source of food	
e14_6_f1	m. MILK AND MILK PRODUCTS	false

e14_6_f1_times_yesterday	How many of times yesterday (past 24 hours)?	
e14_6_f1_source	Main source of food	
e14_7_f1	n. OILS AND FATS	false
e14_7_f1_times_yesterday	How many of times yesterday (past 24 hours)?	
e14_7_f1_source	Main source of food	
e14_7_f2	o. RED PALM PRODUCTS	false
e14_7_f2_times_yesterday	How many of times yesterday (past 24 hours)?	
e14_7_f2_source	Main source of food	
e14_8_f1	p. SUGAR-SWEETS	false
e14_8_f1_times_yesterday	How many of times yesterday (past 24 hours)?	
e14_8_f1_source	Main source of food	
e16_how_long_after_birth_put_to_breast	E 16. How long after birth did you/mother first put (NAME) to the breast?	
e19_still_breastfeeding	E 19. Is (NAME) still breastfeeding currently?	
e20_breastfeeding_stopped_at	E 20. If (NAME) is not still breastfeeding, at what age in months did you stop?	
e21_times_of_breastfeeding	E 21. Last night, how many times did you breastfeed (NAME) from sunset to sunrise?	
e23_anything_dink_in3days_after_delivery	E 23. In the first 3 days after delivery, was [NAME] given anything to drink other than breast milk?	
e24_list_reported	E 24. If yes tick ALL items that are reported. Simply record all liquids mentioned. Do not read the list of possible responses	
e26_a	A Plain water?	
e26_a_times	HOW MANY TIMES/INSHURO	
e26_b	B Infant formula such as [Nan, Guigoz, Nido, etc]?	
e26_b_times	HOW MANY TIMES/INSHURO	
e26_c	C Milk such as tinned, powdered, or fresh animal milk?	
e26_c_times	HOW MANY TIMES/INSHURO	
e26_d	D Juice or juice drinks?	
e26_d_times	HOW MANY TIMES/INSHURO	
e26_e	E Clear broth?	
e26_e_times	HOW MANY TIMES/INSHURO	
e26_f	F Yogurt?	
e26_f_times	HOW MANY TIMES/INSHURO	
e26_g	G Thin porridge?	
e26_g_times	HOW MANY TIMES/INSHURO	
e26_h	H Any other liquids such as [inzoga, ikigage, etc]?	
e27_how_many_times_eat_other_than_liquids	E 27. How many times did [NAME] eat solid, semi-solid or soft foods other than liquids yesterday during the day or at night? (question for children >6 months)	

e28_1	a. Bread, rice, noodles/makaroni, or other foods made from grains, including thick grain-based porridge?	
e28_2	b. White potatoes, white yams/amateke ibikoro, manioc, cassava, or any other foods made from roots?	
e28_3	c. Pumpkin, squash, carrots, or sweet potatoes that are yellow or orange inside?	
e28_4	d. Any foods made from beans, peas, lentils or nuts, including Plumpy 'nut?	
e28_5	e. Any dark green leafy vegetables?	
e28_6	f. Any other fruits or vegetables?	
e28_7	g. Any red meat such as beef, pork, lamb, goat, chicken or duck?	
e28_8	h. Any white meat such as chicken or duck or other birds?	
e28_9	i. Fresh or dried fish, or seafood?	
e28_10	j. Eggs?	
e28_11	k. Cheese, yogurt, or milk, and other milk products?	
e28_12	l. Any oil, fats or butter, or foods made with any of these?	
e28_13	m. Any sugary foods such as chocolates, sweets, candies, pastries, cakes or biscuits?	
e28_15	o. Any milk/animal source food from own farm?	
e16_17_any_plumpy_nut	q. Any plumpy nut?	
e29_who_prepated_meals	E 29. During the last two weeks, who prepared most of the meals for the child?	
e31_times_child_fed_by_others	E 31. During the last two weeks, how many days was the child fed by someone other than mother and father?	
e33_given_vitamina_capsule	E 33. Was your baby given any vitamin A capsule in the last 6 months?	
e34_received_deworming_tablets	E 34. Has (name of the child) received deworming tablets in the last 6months	
e35_ b used_multiple_micronutrient_powder	E 35 b. Have you used multiple micronutrient powder to add to the foods of (name of the child) in the last week?	
e37_received_plumpy_nut	E 37. Did the child receive plumpy nut /RUTF (Ready to Use Therapeutic Food) in the last 3 months?	
e37_b	E 37b. Did the child receive fortified food (Shishakibondo) in the last 2 weeks?	
e38_ever_attended_nutrition_clinic	E 38. Did your child ever attend a nutrition clinic like Supplementary feeding programme or Therapeutic feeding centre?	
e40_nutrition_education_in_village	E 40. Is there any nutrition education and counselling program in the community (village)?	
e41_you_attend_any_nutrition_education	E 41. In the last month, did you attend any session on nutrition education and counselling	
e44_times_of_growth_monitoring	E 44. How many times did your child receive growth monitoring measurement in the last 6 months?	
e45_received_nutrition_support	E 45. Have you ever received any support in nutrition program?	

e46_type_of_received_services	E 46. If Yes, what type of services did you receive?	
e47_hh_livestock	E 47. Does the household keep livestock?	
e47_hh_livestock_type	If yes, what types of livestock do you keep?	
e48_hh_have_home_garden	E 48. Does the household have a home garden	
e49_type_of_vegetables_in_garden	E 49. What types of vegetables and nutrient dense crops (bio-fortified crops) are grown in the field	
e49_a	E 49.a. Which of the following nutrient dense crops has your household ever grown?	
e50_type_of_fruit_trees	E 50. What type of fruit trees are grown by the household?	
e51_how_use_garden_products	E 51. How do you use the kitchen/home garden produces?	
e52_main_source_food_household_consumption	E 52. How do you get access to the food that your household consumes?	
f1_farm_size	F 1. What is your average farm size owned for agricultural activities?	
f2_livestock	F 2. What types of livestock do you keep?	
f3_milk_product	F 3. What type of milk product do you take at your house?	
f4_make_fermented_milk	F 4. If you take fermented milk, how do you make it?	
f5_milk_teatment_before_consumption	F 5. What type of (milk) treatment do you perform before consumption?	
f6_boiling_duration	F 6. How long do you boil milk before drinking?	
f7_keeping_milk_before_boiling_duration	F 7. How long do you keep the milk before boiling?	
f8_boiled_milk_container	F 8. In which type of containers do you keep boiled milk?	
g1_1_prevents_seeing_friends	a. He tries to keep you from seeing your friends	
g1_3_restricts_contact_family	b. He tries to restrict contact with your family of birth	
g1_5_ignores_treats_indifferntly	c. He ignores you and treats you indifferently	
g1_7_gets_angry_speaking_another_man	d. He gets angry if you speak with another man	
g1_9_suspicious_that_unfaithful	e. He is often suspicious that you are unfaithful	
g1_11_expects_permission_for_health_care	f. He expects you to ask his permission before seeking health care for yourself	
g1_13_controls_how_spend_money	g. He controls how you spend your money	
g2_a_a	a. Insulted you or made you feel bad about yourself	
g2_a_b	-Did this happen at any time in your life before the pregnancy period with the child NAME?	
g2_a_c	-Did this happen to you during the pregnancy or after your child NAME was born?	
g2_b_a	Belittled or humiliated you in front of other people?	
g2_b_b	-Did this happen in the year before the pregnancy with the child NAME?	
g2_b_c	-Did it happen to you during the pregnancy or after your child NAME was born?	
g2_c_a	Done things to scare or intimidate you on purpose?	
g2_c_b	-Did this happen in the year before the pregnancy with the child NAME?	
g2_c_c	-Did this happen to you during the pregnancy or after your child NAME was born?	
g2_d_a	Threatened to hurt someone you care about?	
g2_d_b	-Did this happen in the year before the pregnancy with the child NAME?	
g2_d_c	-Did this happen to you during the pregnancy or after your child NAME was born?	
g2_more	More information to add related to above	

g3_a_a	a. Slapped you or thrown something at you that could hurt you?	
g3_a_b	-Did this happen at any time in your life before the pregnancy period with the child NAME?	
g3_a_c	-Did this happen to you during the pregnancy or after your child NAME was born?	
g3_b_a	b. Pushed you or shoved you?	
g3_b_b	-Did this happen at any time in your life before the pregnancy with the child NAME?	
g3_b_c	-Did this happen to you during this pregnancy period or after your child NAME was born?	
g3_c_a	c. Hit you or shoved you?	
g3_c_b	-Did this happen at any time in your life before the pregnancy with the child NAME?	
g3_c_c	-Did this happen to you during this pregnancy period or after your child NAME was born?	
g3_d_a	d. Kicked you, dragged you or beaten you up?	
g3_d_b	-Did this happen at any time in your life before the pregnancy with the child NAME?	
g3_d_c	-Did this happen to you during this pregnancy period or after your child NAME was born?	
g3_e_a	e. Choked or burnt you on purpose?	
g3_e_b	-Did this happen at any time in your life before the pregnancy with the child NAME?	
g3_e_c	-Did this happen to you during this pregnancy period or after your child NAME was born?	
g3_f_a	f. Threatened to use or actually used a gun, knife or other weapon against you?	
g3_f_b	-Did this happen at any time in your life before the pregnancy with the child NAME?	
g3_f_c	-Did this happen to you during this pregnancy period or after your child NAME was born?	
g3_more	More information to add related to above	
g4_a_a	a. Did your current or former husband/partner ever physically force you to have sexual inter-course when you did not want to?	
g4_a_b	-Did this happen at any time in your life before the pregnancy period with the child NAME?	
g4_a_c	-Did this happen during this pregnancy period or after your child NAME was born?	
g4_b_a	Did you ever have sexual intercourse you did not want to because you were afraid of what your current or former husband/partner might do?	
g4_b_b	-Did this happen at any time in your life before the pregnancy with the child NAME?	
g4_b_c	-Did this happen during this pregnancy period or after your child NAME was born?	
g4_c_a	Did your current or former husband/partner ever force you to do something sexual that you found degrading or humiliating?	
g4_c_b	-Did this happen at any time in your life before the pregnancy with the child NAME?	
g4_c_c	-Did this happen to you during this pregnancy period or after your child NAME was born?	
g5_violence_from_others_during_pregnancy	G 5. Has any other person than your husband used physical or sexual violence towards you during your latest pregnancy?	
g6_who	G 6. If 'YES' to the above question, who was this person?	

g7_1_had_cuts_bruises_aches	a. You had cuts, bruises, or aches?	
g8_afraid_of_husband	G 8. Are you afraid of your (last) husband/partner?	
g10_1	1. Took away privileges, forbade something the he/she/Name liked, or did not allow him/her/Name to leave the house (child NAME).	
g10_2	*2. Did this happen to any other child/children in the family?*	
g10_5	5. Shook him/her (NAME of the child NAME).	
g10_6	*6. Did this happen to any other child/children in the family?*	
g10_7	7. Shouted, yelled at or screamed at him/her/Name (child NAME).	
g10_8	*8. Did this happen to any other child/children in the family?*	
g10_9	9. When he/she/NAME did something wrong, did you or any other adult in the household give him/her/NAME something else to do?	
g10_10	*10. Did this happen to any other child/children in the family?*	
g10_11	11. Hit him/her on the bottom with bare hand	
g10_12	*12. Did this happen to any other child/children in the family?*	
g10_13	13. Hit him/her on the bottom or elsewhere on the body with something like a belt, hairbrush, stick or other hard object (child NAME/Name).	
g10_14	*14. Did this happen to any other child/children in the family?*	
g10_15	15. Called him/her dumb, lazy or another name like that (child NAME/Name).	
g10_16	*16. Did this happen to any other child/children in the family?*	
g10_21	21. Beat him/her up, that is hit him/her over and over as hard as one could (child NAME/Name).	
g10_22	*22. Did this happen to any other child/children in the family?*	
g11_do_believe_in_children_physical_punishment	G 11. Do you believe that in order to bring up, raise, or educate a child properly, the child needs to be physically punished?	
h1_today_overal_health	H 1. Would you say your overall health today is:	
h2_6	6. Anxiety	
h2_7	7. Depression	
h2_8	8. Headache	
h2_9	9. Fatigue, extreme tiredness	
h3_1	1. Tuberculosis	
h3_2	2. HIV/AIDS	
h3_3	3. Malaria	
h3_4	4. Low body weight, being too slim	
h3_5	5. High blood pressure	
h3_13	13. Diarrhoea	
h3_14	14. Intestinal worms	
h3_20_other	Any other disease, please specify:	
h4_somke_before_pregnancy	H 4. Before the pregnancy with the child NAME, did you smoke tobacco? If Yes please indicate how many times do you smoke per day.	
h5_smoke_during_pregnancy	H 5. Did you smoke any cigarettes during the pregnancy with the child NAME? If Yes please indicate how many times do you smoke per day	

h6_alcohol_before_pregnancy	H 6. Did you use any form of alcohol before the pregnancy with the child NAME?	
h7_alcohol_during_pregnancy	H 7. Did you use any form of alcohol during the pregnancy with the child NAME?	
h9_how_often_husband_drinks	H 9. How often does your husband/partner drink alcohol?	
h_a1	*A1. Have you been consistently depressed or down, most of the day, nearly every day, for the past two weeks?*	
h_a2	*A2. In the past two weeks, have you been less interested in most things or less able to enjoy the things you used to enjoy most of the time?*	
h_a3_a	a. Was your appetite decreased or increased nearly every day or did your weight decrease or increase without trying intentionally?	
h_a3_b	b. Did you have trouble sleeping nearly every night (difficulty falling asleep, waking up in the middle of the night, early morning wakening, or sleeping excessively)?	
h_a3_c	c. Did you talk or move more slowly than normal or were you fidgety, restless or having trouble sitting still, almost every day?	
h_a3_d	d. Did you feel tired or without energy, almost every day?	
h_a3_e	e. Did you feel worthless or guilty, almost every day?	
h_a3_f	f. Did you have difficulty concentrating or making decisions, almost every day?	
h_a3_g	g. Did you repeatedly consider hurting yourself, feel suicidal, or wish that you were dead?	
h_a4	A4. MAJOR DEPRESSIVE EPISODE CURRENT	
h_a5_a	A5 a. During your lifetime, did you have other periods of two weeks or more when you felt depressed or uninterested in most things, and had most of the problems we just talked about?	
h_a5_b	A5 b. Was there an interval of at least 2 months without depression and/or lost of interest between your current episode and your last episode of depression?	
h_a5	A5. MAJOR DEPRESSIVE EPISODE PAST	
h_b1	B1. Do you think that you would be better off dead or wish you were dead?	
h_b2	B2. Do you want to harm yourself?	
h_b3	B3. Do you think about suicide?	
h_b4	B4. Do you have a suicide plan?	
h_b5	B5. Do you attempt suicide?	
h_b6	B6. In your life time, did you ever make a suicide attempt?	
h_b	SUICIDE RISK CURRENT	
h_c1_a	*C1 a. Have you worried excessively or been anxious about several things of day to day life, at work, at home, in your close circle over the past 6 months?*	
h_c1_b	*C1 b. Are these worries present most days?*	

h_c2	C2. Do you find it difficult to control the worries or do they interfere with your ability to focus on what you are doing?	
h_c3_a	a. Feel restless, keyed up or on edge?	
h_c3_b	b. Feel tense?	
h_c3_c	c. Feel tired, weak or exhausted easily?	
h_c3_d	d. Have difficulty concentrating or find your mind going blank?	
h_c3_e	e. Feel irritable?	
h_c3_f	f. Have difficulty sleeping (difficulty falling asleep, waking up in the middle of the night, early morning wakening or sleeping excessively)?	
h_c3	GENERALIZED ANXIETY DISORDER CURRENT	
i1_decision_major_purchases	I 1. Who usually makes decisions about making major household purchases?	
i2_decision_visit_family	I 2. Who usually makes decisions about visits to your family, relatives and friends?	
i3_decision_on_earned_money	I 3. Who usually decides how the money you earn will be used?	
i4_own_house	I 4. Do you own this or any other house either alone or jointly with someone else?	
i5_own_any_land	I 5. Do you own any land either alone or jointly with someone else?	
i6_who_make_decision_abou_health	I 6. Who usually makes decisions about health care for yourself?	
i7_influences_on_family_planning	I 7. Who or what influences your decision on family planning the most?	
i8_can_say_no_to_sexual_intercourse	I 8. Can you say no to your husband/partner if you do not want to have sexual intercourse?	
i9_can_ask_use_candom	I 9. Could you ask your (husband/partner) to use a condom if you wanted him to?	
j_01	Capture a photograph of the "immunisation card document", "house", or other important documents.	
j_02	If you need, you could record a short interview (less than five minutes).	
k1_land_ownership	K 1. Type of land ownership	
k3_watering_livestock	K 3. Is water for watering livestock available in the area?	
k4_water_sources	K 4. Sources of water available in the area	
k5	K 5. Frequency of offering water to animals	
k6	K 6. If “No”, why?	
k7_month_water_livestock	K 7. Which months do you experience water shortage for watering your livestock (including fodder production)?	
k8_annual_water_quality	K 8. What is water quality used in your farm?	
k9_water_quality_test	K 9. How do you test the quality of water?	
k10_livestock _feed_shortage	K 10. Do you experience feed shortage for your livestock?	
k11	K 11. If “Yes” why?	
k12_month_feed_shortage	K 12. Indicate which months do you experience feed shortage?	
k13	K 13. Availability of mineral block	
k14	K 14. If “Yes”, where did you get it	
k15	K 15. If “No”, why?	
k16_purchase_feed	K 16. Do you purchase feed?	
k17_feed_purchase_types	K 17. If yes, indicates the major type of feed purchased per year.	
k18_annually_feed_purchased	K 18. Indicate the cost of feed purchased annually?	

k19_planted_forages_fodder	K 19. Do you have planted forages/fodder?	
k20_farm_crops	K 20. If Yes, indicate types of crops grown in your farm	
k21_forage_decision_maker	K 21. Who makes decision on the type of forage to plant/grow?	
k22_forage_place_in_farm	K 22. Where in your farm do you plant your forages? (several response alternatives possible)	
k23_forage_area_dictator	K 23. What dictates the area you plant fodder/forages?	
k24_fertilizer_forages	K 24. Do you apply fertilizer to your forages	
k25_fertilizer	K 25. If yes which one?	
k26_f18_why_not	K 26. If no why not?	
k27_feed_conserve_type	K 27. How do you conserve feed for your livestock?	
k28	K 28. Who are involved in grass hay, silage production?	
k29_why_not_forages	K 29. Why don't you have planted forages?	
k30_crop_residues_used_for	K 30. Uses of crop residues from the farm	
k31	K 31. If fed to livestock, how do you treat the crop residues for quality improvement?	
k32	K 32. If you use urea, what are the impact in livestock production?	
k33_collect_fodder_for_livestock	K 33. Do you collect or gather green fodder to feed your livestock	
k34_other_fodder_benefits	K 34. If “NO”, list other benefit(s) of green fodder.	
k35_processed_feed_for_livestoc k	K 35. Do you use homemade concentrates for livestock in your farm?	
k36_f26_what_feed	K 36. If “YES”, what feed do they use?	
k37_f26_why_no	K 37. If No, why no?	
k38_keeping_record_at_farm	K 38. Do you keep record at your farm?	
k39_what_records	K 39. If 'YES' what records do you keep?	
k40_farming_system	K 40. What farming system are you practicing?	
k41_reproduction_technique	K 41. What type of reproduction technique do you use in your farm?	
k42_get_mating_bull_from	K 42. If natural mating. Where do you get the bull?	
k35_have_land_for_forage	K 35. In case of extensive system. Do you have enough land for forage production?	
k43_animal_house_floor	K 43. Type of animal house floor	
k44_feeding_style	K 44. What is the style of feeding?	
k45	K 45. If open grazing, where do you graze your animal?	
k46_animal_house_cleaning_peri od	K 46. How often do you clean the animal house?	
k47_milking_cows_number	K 47. How many milking cows do you have?	
k48_milking_times_per_day	K 48. How many times per day do you milk?	
k49_milking_technique	K 49. What milking technique do you uses?	
k50_produced_ltrs_per_farm_per _day	K 50. How many liters do you produce per farm per day?	
k51_lactation_period	K 51. For how long do your cows produce milk per lactation?	
k52_how_proceed_milking	K 52. How do you proceed when milking?	
k53_milking_containers	K 53. Which type of milking containers do you use?	
k54_where_do_milk	K 54. Where do you milk from?	
k55_do_screen_subclinical_masti tis	K 55. Do you screen for subclinical mastitis?	
k56_how_do_ensure_milk_quality	K 56. How do you ensure milk quality in your herd? (several response alternatives possible)	
k57_milk_production_purpose	K 57. What is the main purpose of your milk production?	
k58_where_do_sell_milk_product	K 58. Where do you sell your milk produce?	
k59_treatment_before_consumpti on	K 59. What type of (milk) treatment do you perform before consumption?	
k60_howlong_boil_milk	K 60. How long do you boil milk before drinking?	
k61_when_do_wash_containers	K 61. When do you wash milking containers?	

k62_water_source_for_cleaning_utensils	K 62. From where do you get water for cleaning milking utensils?	
k63_water_type_for_cleaning_equipment	K 63. What type of water do you use to clean milking equipment?	
k64_howlong_to_deliver_milk	K 64. How long does it take you to deliver milk to your client(s)?	
k65_container_type_for_transportation	K 65. Which type of containers do you use for milk transportation?	
k66_means_of_transport	K 66. What means of transport do you use to deliver your milk produce?	
k67_experienced_milk_rejection	K 67. Did you ever experience milk rejection?	
k68_rejection_reasons	K 68. If yes, what were reasons for milk rejection?	
k69_what_do_with_rejected_milk	K 69. What do you do with the rejected milk?	
k70_diseases_experienced_in_herds	K 70. What are the animal diseases do you experience in your herd(s)?	
k71_reproductive_diseases_in_herds	K 71. What reproductive diseases do you experience in your herd?	
k72_do_know_animal_human_disease_transmit	K 72. Do you know that animals may transmit diseases to humans?	
k73_how_think_transmit	K 73. If yes, how do you think this transmission may occur?	
k74_which_disease_think_can_transmit_human	K 74. Which animal disease do you think can be transmitted to humans? (Several response alternatives possible)	
k75_how_prevent_these_disease	K 75. How can these disease be prevented in humans? (Several response alternatives possible)	
k76	K 76. Have any household member had any gastrointestinal symptoms during the last 2 weeks?	
k77	K 77. If “yes” what kind of symptoms?	
k78	K 78. Do you know what caused these symptoms?	
k79	K 79. If “yes” what are the causes?	
k80_what_prevention_mechanisms_know	K 80. What are the mechanisms of disease prevention in animals do you know? (Several response alternatives possible)	
k81_how_newanimals_introduced_herd	K 81. How are new animals introduced in the herd?	
k81_1_quarantine_days	If quarantine, specify the duration in days	
k81_2_what_do_to_animals_in_quarantine	If quarantine, what do you do to the animals during that time?	
k82_where_get_veterinary_services	K 82. Where do you get veterinary services from (when your animals are sick)?	
k83_what_do_chronic_sick_animals	K 83. What do you do when you have a chronic sick animal?	
k84_what_do_with_milk_from_sickanimals	K 84. What do you do with the milk from sick animals (under treatment)?	
k85_previous_animal_keeping_experience	K 85. Did you or any household member have previous experience in animal keeping?	
k86_had_training_farming_production_management	K 86. Have you had any training on farming production management?	
k87_what_module_trained	K 87. If yes, what module(s) have you been trained on?	
k88_improvement_done_after_trainings	K 88. What improvement did you make after these trainings?	
insertedat	Inserted at	
insertedbyusername	Inserted by	
updatedat	Updated at	
updatedbyuserid	Updated by UserId	
updatedbyusername	Updated by	
record_insertion_uid	record_insertion_uid	
