# Supplementary material for: Identification of amendable risk factors for childhood stunting at individual, household and community levels in Northern Province, Rwanda – a cross-sectional population-based study
Source: BMC Public Health. 2025 Mar 21;25:1087. doi: 10.1186/s12889-025-22329-8 (PMC11927283; doi:10.1186/s12889-025-22329-8)
Supplement: Supplementary file 2 — Supplementary Material 2 [file 12889_2025_22329_MOESM2_ESM.docx]

Additional file 1. Description of the household characteristics and bivariate analysis for the relationship with childhood stunting, Northern Province, 2021 (N=601)

| **Variables** | **Total** | **No stunting** | **Stunting** | **COR** | | **(95%CI)** | | **p-value** | |
| --- | --- | --- | --- | --- | --- | --- | --- | --- | --- |
|  |  | **n (%)** | **n (%)** |  | |  |  |  |  |
| ***Home environment characteristics*** | | | | | | | | |  |
| Childcare practices | | | | | | | | |  |
| *Child left alone more than 1 hour in last 15 days (No=reference)* | | | | |  | |  | |  |
| Yes | 195 (32.4) | 135 (69.2) | 60 (30.8) | 1.31 | | 0.90,1.91 | | 0.164 | |
| *Child left in care of another child last week (No=reference)* | | | | | | | | | |
| Yes | 176 (29.3) | 125 (71.0) | 51 (29.0) | 1.14 | | 0.77,1.69 | | 0.510 | |
| *Who prepared the meal for the child in the last 15 days* | | | | | | | | | |
| Biological mother | 524 95.3) | 381 (72.71) | 143 (27.29) | 1 | |  | |  | |
| Others | 26 (4.7) | 16 (61.54) | 10 (38.46) | 1.67 | | 0.74,3.75 | | 0.219 | |
| *How many days was the child fed by someone other than mother and father in last 2 weeks* | | | | | | | | | |
| Never | 325 (54.1) | 254 (78.15) | 71 (21.85) | 1 | |  | |  | |
| 1 day or more | 276 (45.9) | 184 (66.67) | 92 (33.33) | 1.79 | | 1.24, 2.57 | | 0.002 | |
| *Time to breast after birth (n=594)* | | | | |  | |  | |  |
| Within 1 hour | 525 (88.7) | 393 (74.9) | 132 (25.1) | 1 | |  | |  | |
| After 1 hour and above | 67 (11.3) | 40 (59.7) | 27 (40.0) | 2.01 | | 1.19, 3.40 | | 0.009 | |
| *Child still breastfeeding at the time of interview (n=594, yes=reference)* | | | | |  | |  | |  |
| No | 142 (23.9) | 87 (61.3) | 55 (38.7) | 2.01 | | 1.40, 3.12 | | <0.001 | |
| Household socioeconomic characteristics | | | | | | | | |  |
| *Mother societal support (n=593, Yes=reference)* | | | | |  | |  | |  |
| No | 363 (61.2) | 247 (68.04) | 116 (31.96) | 1.88 | | 1.27, 2.78 | | 0.002 | |
| *Twin children aged 1-36 months in the household (No=reference)* | | | | |  | |  | |  |
| Yes | 39 (6.5) | 20 (51.3) | 19 (48.7) | 2.76 | | 1.43, 5.31 | | 0.002 | |
| *Mother's education(n=543)* | | | | |  | |  | |  |
| Less than primary | 219 (40.3) | 168 (76.7) | 51 (23.3) | 1 | |  | |  | |
| Primary level | 193 (35.5) | 143 (74.1) | 50 (25.9) | 1.15 | | 0.73, 1.81 | | 0.538 | |
| Secondary or higher | 131 (24.1) | 91 (69.5) | 40 (30.5) | 1.45 | | 0.89, 2.35 | | 0.136 | |
| *Ubudehe category* | | | | |  | |  | |  |
| Ubudehe 1 | 68 (11.3) | 509 (73.53) | 18 (26.47) | 1.06 | | 0.58, 1.96 | | 0.847 | |
| Ubudehe 2 | 292 (48.6) | 208 (71.23) | 84 (28.77) | 1.19 | | 0.81, 1.75 | | 0.372 | |
| Ubudehe 3 | 241 (40.1) | 180 (74.69) | 61 (25.31) | 1 | |  | |  | |
| *Sex of Household head (n=600)* | | | | |  | |  | |  |
| Male | 553 (65.8) | 410 (74.1) | 143 (25.9) | 1 | |  | |  | |
| Female | 47 (5.6) | 27 (57.4) | 20 (42.6) | 2.12 | | 1.16, 3.90 | | 0.015 | |
| Intrahousehold food allocation | | | | | | | | |  |
| *Have milking cow (Yes=reference)* | | | | |  | |  | |  |
| No | 445 (47.0) | 316 (71.0) | 129 (29.0) | 1.46 | | 0.95, 2.26 | | 0.083 | |
| *Kitchen garden (Yes=reference)* | | |  |  | |  | |  | |
| No | 153 (25.5) | 100 (65.4) | 53 (34.6) | 1.63 | | 1.10, 2.42 | | 0.016 | |
| *Main source of food for the Household* | | | | |  | |  | |  |
| Harvest & market | 525 (92.8) | 392 (74.67) | 133 (25.33) | 1 | |  | |  | |
| Market | 35 (6.2) | 20 (57.14) | 15 (42.86) | 2.21 | | 1.10, 4.44 | | 0.026 | |
| Harvest | 6 (1.1) | 4 (66.67) | 2 (33.33) | 1.47 | | 0.27, 8.14 | | 0.656 | |
| *Household Food Insecurity Access (n=597)* | | | | |  | |  | |  |
| Total score (SD) | 10.12 (7.18) | 9.53 (7.10) | 11.7 (7.17) | 1.04 | | 1.02, 1.07 | | 0.001 | |
| Food secure | 74 (12.4) | 64 (86.5 | 10 (13.5) | 1 | |  | |  | |
| Mildly Food Insecure | 50 (8.4) | 38 (76.0) | 12 (24.0) | 2.02 | | 0.80, 5.12 | | 0.138 | |
| Moderately Food Insecure | 212 (35.5) | 156 (73.6) | 56 (26.4) | 2.30 | | 1.10, 4.78 | | 0.026 | |
| Severely Food Insecure | 261 (43.7) | 177 (67.8) | 84 (32.2) | 3.04 | | 1.49, 6.21 | | 0.002 | |
| Sanitation and water supply | | | | | | | | |  |
| *Type of toilet* | | | | |  | |  | |  |
| Improved | 272 (45.3) | 209 (86.8) | 63 (23.2) | 1 | |  | |  | |
| Unimproved | 329 (54.7) | 229 (69.6) | 100 (30.4) | 1.45 | | 1.00, 2.09 | | 0.048 | |
| *Handwashing station within 5 meters from the toilet (Yes=reference)* | | | | |  | |  | |  |
| No | 532 (88.5) | 376 (70.7) | 156 (29.3) | 3.67 | | 1.65, 8.21 | | 0.001 | |
| *Washed hands with soap in the last 24 hours (Yes=reference)* | | | | |  | |  | |  |
| No | 84 (14.0) | 52 (61.9) | 32 (38.1) | 1.82 | | 1.12, 2.95 | | 0.015 | |
| *Handwashing at critical moments (Yes=reference)* | | | | |  | |  | |  |
| No | 171 (28.5) | 112 (65.50) | 59 (34.50) | 1.65 | | 1.12, 2.43 | | 0.011 | |
| *Treat water to drink (n=600, Yes=reference)* | | | | |  | |  | |  |
| No | 327 (54.5) | 234 (71.6) | 93 (28.4) | 1.17 | | 0.82, 1.69 | | 0.385 | |
|  |  |  |  |  | |  | |  | |
| ***Societal/community characteristics*** | | | | | | | | | |
| *Time to water source (Go. queue and return) (n=581)* | | | | | | | | | |
| Water on premises | 302 (52.0) | 220(72.8) | 82(27.2) | 1 | |  | |  | |
| Less than 30 minutes | 44 (7.6) | 33(75.0) | 11(25.0) | 0.89 | | 0.43,1.85 | | 0.764 | |
| 30 minutes and above | 235 (40.4) | 170(72.3) | 65(27.7) | 1.03 | | 0.70,1.50 | | 0.896 | |
| *Travel time to the nearest health facility* | | | | | | | | | |
| <0:30 minutes | 108 (18.4) | 78(72.2) | 30(27.8) | 1 | |  | |  | |
| 0:30-0:59 minutes | 167 (28.5) | 121(72.5) | 46(27.5) | 0.99 | | 0.58,1.70 | | 0.966 | |
| 1H00-1H30 | 218 (37.2) | 157(72.0) | 61(28.0) | 1.01 | | 0.60,1.69 | | 0.969 | |
| >1H30 | 93 (15.9) | 71(76.3) | 22(23.7) | 0.81 | | 0.43,1.52 | | 0.506 | |
| *Travel distance to the nearest health facility* | | | | | | | | | |
| <1 km | 136 (22.6) | 104(76.5) | 32(23.5) | 1 | |  | |  | |
| 2.9 km | 195 (32.4) | 146(74.9) | 49(25.1) | 1.09 | | 0.65,1.82 | | 0.739 | |
| ≥3km | 270 (44.9) | 188(69.6) | 82(30.4) | 1.42 | | 0.88,2.28 | | 0.149 | |
| *Child received Shishakibondo^1^ in last 2 weeks (Yes=reference)* | | | | | | | | | |
| No | 525 (87.4) | 383(73.0) | 142(27.1) | 0.97 | | 0.57,1.66) | | 0.915 | |
| *Existence of nutrition education and counselling program in the village (Yes=reference)* | | | | | | | | | |
| No | 199 (33.1) | 145(72.9) | 54(27.1) | 1.00 | | 0.68,1.47 | | 0.996 | |
| *Attending nutrition education and counselling sessions (Yes=reference)* | | | | | | | | | |
| No | 348 (57.9) | 258(74.1) | 90(25.9) | 0.86 | | 0.60,1.24 | | 0.416 | |
| *Child vaccinated (n=600, Yes=reference)* | | | | | | | | | |
| No | 10 (1.7) | 8(80.0) | 2(20.0) | 0.66 | | 0.14,3.17 | | 0.610 | |
| *Elevation (m)* |  |  |  |  | |  | |  | |
| Mean (SD) | 1962.7(242.6) | 1958.2(240.0) | 1974.6(249.8) | 1.00 | | 0.99, 1.00 | | 0.462 | |
| 1400-1899.9 | 273 (45.4) | 203(74.4) | 70(25.64) | 1 | |  | |  | |
| 1900-2761 | 328 (54.6) | 235(71.7) | 93(28.35) | 1.15 | | 0.80,1.65 | | 0.457 | |

^1^ A mix of maize flour, vitamins and minerals given as part of nutritional support.
